# Early duodenal adenocarcinoma resembling a submucosal tumor cured with endoscopic resection: a case report

**DOI:** 10.1186/1752-1947-6-280

**Published:** 2012-09-04

**Authors:** Akira Dobashi, Kenichi Goda, Noboru Yoshimura, Kazuki Sumiyama, Hirobumi Toyoizumi, Shoichi Saito, Tomohiro Kato, Hiroki Ishikawa, Katsuhiko Yanaga, Hisao Tajiri, Masahiro Ikegami

**Affiliations:** 1 Department of Endoscopy, The Jikei University School of Medicine, 3-25-8, Nishi-shimbashi, Minato-ku, Tokyo, 105-8461, Japan; 2 Minato Mirai Medical Square Clinic, 3-6-3, Minato Mirai, Nishi-ku, Yokohama, 220-012, Japan; 3 Department of Surgery, The Jikei University School of Medicine, 3-25-8, Nishi-shimbashi, Minato-ku, Tokyo, 105-8461, Japan; 4 Division of Gastroenterology and Hepatology, Department of Internal Medicine, The Jikei University School of Medicine, 3-25-8, Nishi-shimbashi, Minato-ku, Tokyo, 105-8461, Japan; 5 Department of Pathology, The Jikei University School of Medicine, 3-25-8, Nishi-shimbashi, Minato-ku, Tokyo, 105-8461, Japan

## Abstract

**Introduction:**

Primary adenocarcinomas resembling submucosal tumors are rare in the gastrointestinal tract. Almost all the submucosal tumor-like adenocarcinomas previously reported invaded the submucosa or deeper. Therefore, submucosal tumor-like lesions are usually treated by surgical resection, and those that arise in the duodenum have been treated by pancreaticoduodenectomy.

**Case presentation:**

A 65-year-old Japanese man was diagnosed with a submucosal tumor-like adenocarcinoma in his duodenum. We considered it possible that the tumor invasion was limited to the mucosal or submucosal layers and could be removed by endoscopic resection. Tumor histopathology revealed a well-differentiated adenocarcinoma confined to the muscularis mucosae with no lymphovascular invasion. Complete resection of the carcinoma was achieved and there has been no recurrence three years after endoscopic resection.

**Conclusions:**

We suggest that submucosal tumor-like adenocarcinomas arising in nonampullary duodenal sites should be diagnosed carefully with a view to possible endoscopic resection.

## Introduction

Adenocarcinomas are epithelial-derived tumors while submucosal tumors (SMTs) originate from nonepithelial cells. Hence, there have been few reports of adenocarcinoma lesions resembling SMTs in the digestive tract. Previous reports have described SMT-like adenocarcinomas in the stomach and colon [1-5]. However there have been no reports in the English or Japanese literature of adenocarcinoma lesions resembling SMTs in the duodenum. All of the previously reported SMT-like lesions, except for one report, were treated by surgical resection (SR).

We report a rare case of duodenal adenocarcinoma resembling a SMT, which was resected completely, not by pancreaticoduodenectomy as originally scheduled, but by endoscopic resection (ER).

## Case presentation

**Figure 1:**
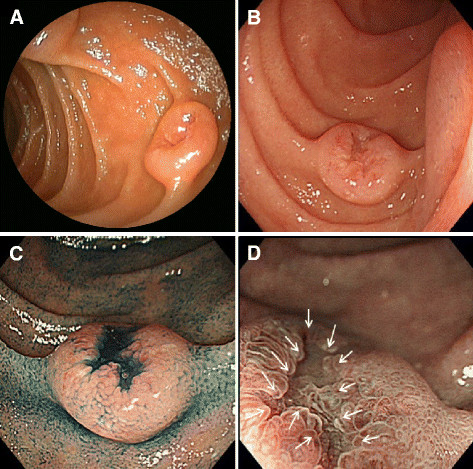
**Endoscopic findings.** ( **A**) Conventional endoscopy before multiple biopsies at a medical checkup in November 2009 showed a submucosal tumor-like polypoid lesion with a central deep depression. ( **B**) In work-up prior to surgery in May 2010, conventional endoscopy showed a submucosal tumor-like polypoid lesion with a depression. The lesion was decreased in height and had tense surface mucosa compared with the lesion seen during the initial esophagogastroduodenoscopy at the medical checkup in November 2009. ( **C**) Chromoendoscopy after spraying indigo carmine solution revealed a marginal portion of the polypoid lesion covered by normal duodenal mucosa. The depressed portion showed an irregular margin. ( **D**) Narrow-band imaging magnified endoscopy revealed an obscure mucosal pattern with irregular microvessels in the depressed portion (arrows).

A 65-year-old Japanese man, with no symptoms and receiving only oral medication for hypertension, underwent an esophagogastroduodenoscopy for a medical checkup in November 2009. A polypoid lesion, about 10mm in diameter with a deep depression on top, was found on the opposite side of the Vater papilla in the second portion of his duodenum (Figure 1A). The lesion was covered by normal mucosa except in the depressed portion, which showed tense surface mucosa. The lesion was similar in appearance to a SMT with a central depression. Multiple biopsies were taken from the lesion, but all of the specimens showed normal duodenal mucosa. The lesion was strongly suspected to be a malignant tumor and six more biopsies were taken from the lesion during a second esophagogastroduodenoscopy three months later. Only one of the six biopsy specimens revealed adenocarcinoma. On endoscopy, the tumor was suspected of invading the submucosa or deeper because of its endoscopic appearance; therefore, SR was indicated and he was referred for surgery at our university hospital in May 2010.

**Figure 2:**
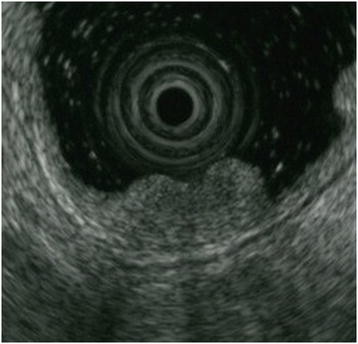
**Endoscopic ultrasonography findings.** Endoscopic ultrasonography (20mHz miniprobe) revealed that the duodenal wall was delineated into five layers. The tumor was visualized as a low echoic mass mainly involving the second or third layers but not the fourth or deeper layers.

Conventional endoscopy prior to surgery showed a polypoid lesion resembling a SMT with a central depression (Figure 1B). Chromoendoscopy after spraying indigo carmine solution revealed an irregular depression at the top of the tumor with its surrounding area covered by normal duodenal mucosa (Figure 1C). Narrow-band imaging (NBI) magnified endoscopy showed an obscure mucosal pattern with irregular microvessels [6], only in the depressed area (Figure 1D). A biopsy specimen from the depressed area revealed adenocarcinoma. High frequency endoscopic ultrasonography (EUS) using a 20mHz miniprobe displayed the duodenal wall as a five-layered structure. The tumor was demonstrated as a low echoic mass mainly involving the second or third layers but not the fourth or deeper layers (Figure 2). Abdominal computed tomography showed no abdominal lymph node swelling. We predicted that it was possible to remove the tumor by ER and the tumor was removed using a conventional endoscopic mucosal resection technique with submucosal injection of glycerin solution and the snare method, instead of by pancreaticoduodenectomy as planned. The endoscopic mucosal resection was performed with no complications and our patient had a straightforward post-ER course. Our patient was discharged from the hospital a week after the ER.

**Figure 3:**
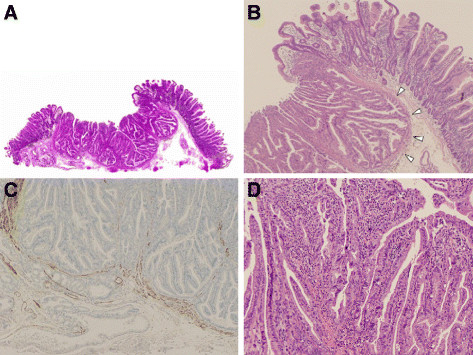
**Histology of endoscopic resection specimen.** ( **A**) Cross-section of the resected specimen (hematoxylin-eosin stain). The tumor surface showed a central depression and bilateral elevations. ( **B**) Dense tumorous glands with papillary growth were seen in the depressed portion. The bilateral elevations were covered by normal duodenal villi. Tumorous glands with an inverted growth downward (arrow heads) led to the bilateral elevations. ( **C**) The tumor invasion was confined to the muscularis mucosae with no submucosal involvement (Desmin stain). ( **D**) High magnification of the tumor illustrated in Figure 3A showing the complex papillary architecture and considerable cytological atypia.

Histopathology of the resected lesion showed a tumor with a central depression and bilateral elevations (Figure 3A). The bilateral elevations were covered by nontumorous glands and pushed up by the tumorous glands showing inverted growth downward (Figure 3B). The tumorous glands invaded expansively to the muscularis mucosae with no submucosal invasion (Figure 3C). Tumor cells showed considerable cytological atypia having abnormal mitosis, heteromorphous nuclei, and eosinophilic cytoplasm, and formed irregular papillary structures (Figure 3D). Histology results from the ER confirmed the tumor was a well-differentiated adenocarcinoma confined to the muscularis mucosae with no lymphovascular invasion and negative margins (Tis N0 M0, Stage 0); complete resection was achieved. Our patient is still alive with no recurrence three years since the tumor was resected by endoscopy.

## Discussion

The prognosis for duodenal adenocarcinoma is reported to be a five-year survival rate of less than 30% [7]. Thus, the prognosis is very poor in patients with an advanced stage [8]. If the duodenal adenocarcinoma is found in its early stage, then endoscopic curative resection is possible [6]. So, it is very important to detect duodenal adenocarcinoma in the early stage.

This case of early duodenal carcinoma showed characteristic features of a SMT on endoscopy. Histopathology demonstrated an inverted growth of cancerous glands with the tumor confined to the muscularis mucosae. We searched reports in English using the key words ‘early carcinoma’, ‘duodenum’, and ‘submucosal tumor-like’ and were unable to find any reports on early duodenal adenocarcinoma resembling SMT. However there were several reports of SMT-like cancer in the stomach and colon, but no reports describing SMT-like adenocarcinomas being completely removed by ER.

According to previous reports of SMT-like cancer in the colon, the deep central depression in these tumors seems to be related to an inverted growth of cancerous glands into the muscularis mucosae, giving the endoscopic appearance of SMT. The histological features of these tumors are similar to an inverted hyperplastic polyp [9]. Repeated mechanical stimulation made by bowel peristaltic contraction has been proposed to cause the distinguishing features of inverted hyperplastic polyps [10]. We consider that inverted growth of a duodenal tumor could be caused by mechanical stimulation because the duodenum has a high peristaltic activity.

Adenocarcinomas resembling SMTs located in the duodenum and colon have the same endoscopic characteristics of precipitously raised lesions with central depressions. A previous report in 2005 reviewed 24 cases of colorectal adenocarcinoma resembling SMT [2]. We found three more cases in previous reports which were published in 2004 to 2007 [3-5], and we examined the clinicopathological features of these 27 lesions in 27 patients (Table 1). All of the tumors invaded the submucosa or deeper, even if the tumor was very small, less than 5mm in diameter. The vast majority of cases (26 of 27) underwent SR. In one case, the carcinoma was resected by endoscopy, however histology showed that the carcinoma had invaded the deep submucosa. Endoscopic findings showed a central depression in most cases (93%). The pathogenesis of SMT-like lesions with a central depression suggests that SMT-like tumors can arise from *de novo* cancer and invade the submucosa, breaking the muscularis mucosae at a very early phase of tumor development. Our case seems to be very rare because the SMT-like tumor was detected before breaking the muscularis mucosae.

**Table 1:** Published cases of carcinoma resembling submucosal tumor in the duodenum and colon

**Case**	**Age (y)**	**Gender**	**Location of lesion**	**Size of lesion (mm)**	**Central depression**	**Depth of cancerous invasion**	**Inverted growth**	**Histologic diagnosis**	**Treatment**
1 [2]	71	M	S	20	present	adv	absent	well	SR
2 [2]	42	F	R	50	present	adv	absent	well	SR
3 [2]	74	F	A	80	present	adv	absent	poor	SR
4 [2]	76	M	A	50	present	adv	absent	mod	SR
5 [2]	62	M	S	10	present	sm	absent	well	SR
6 [2]	44	M	R	80	present	adv	absent	well	SR
7 [2]	44	M	D	50	present	adv	absent	muc	SR
8 [2]	76	M	A	ND	present	adv	absent	mod	SR
9 [2]	64	M	R	10	absent	sm	absent	mod	SR
10 [2]	76	F	R	35	present	adv	absent	muc	SR
11 [2]	60	M	D	15	present	sm	absent	mod	SR after ER
12 [2]	57	M	S	28	present	adv	absent	well	SR
13 [2]	62	M	S	10	present	adv	absent	poor	SR after ER
14 [2]	48	M	T	45	present	adv	absent	poor	SR
15 [2]	53	M	R	14	present	sm	absent	well	SR
16 [2]	48	M	A	15	present	adv	absent	mod	SR
17 [2]	52	M	S	10	present	adv	absent	mod	SR
18 [2]	80	M	A	80	absent	adv	absent	poor	SR
19 [2]	67	M	A	4	present	sm	absent	well	SR after ER
20 [2]	70	M	A	15	present	sm	absent	mod	SR
21 [2]	58	M	S	12	present	adv	absent	well	SR
22 [2]	69	F	C	18	present	sm	absent	mod	SR
23 [2]	70	M	S	14	present	sm	absent	mod	SR
24 [2]	54	M	S	12	present	sm	absent	mod	SR
25 [3]	51	M	S	10	present	sm	present	well	ER
26 [4]	60	M	A	15	present	sm	present	well	SR after ER
27 [5]	62	M	A	40	present	adv	absent	muc	SR
28(our case)	65	M	Duodenum	10	present	mucosae	present	well	ER

A: ascending colon; adv: advanced cancer invading deeper than the muscularis propria; C: cecum; D: descending colon; ER: endoscopic resection; F: female; M: male; mod: moderately-differentiated adenocarcinoma; muc: mucinous carcinoma; N.D: not described; poor: poorly-differentiated adenocarcinoma; R: rectum; S: sigmoid colon; sm: submucosa; SR: surgical resection; T: transverse colon; well: well-differentiated adenocarcinoma.

Mucosal cancers are good candidates for ER because there is no risk of lymph node metastasis [11]. Therefore, it is important to estimate the depth of invasion of duodenal carcinoma and to differentiate mucosal cancer from cancer invading to the submucosal layer or deeper. There are no reports evaluating the depth of invasion in duodenal adenocarcinoma by EUS. However, in colorectal cancers, several studies have evaluated the usefulness of EUS for diagnosing the invasion depth [11-13]. The overall diagnostic accuracy rates of EUS were 75% to 80% [12,13]. One problem with the diagnosis of tumor depth by EUS was overstaging while no understaging was recorded [13]. Thus, in the present case, as EUS demonstrated tumor invasion had not reached the muscularis propria but was only up to the submucosal layer, it had a greater chance of removal by ER. Therefore, we made the decision to remove the tumor by ER in this case mainly based on the EUS findings.

We performed ER as a total biopsy. If histology from the ER had revealed the tumor was confined to the mucosal layer and the horizontal margin was positive, we would have planned an additional ER or endoscopic coagulation therapy. Otherwise, if histology had shown that the tumor had submucosal invasion or lymphovascular invasion, or the vertical margin was positive, we would have planned a partial resection or pancreaticoduodenectomy with lymphadenectomy.

Nakajima and colleagues reported that cancerous glands were evident in the depressed portion of the lesion in most of the SMT-like carcinomas [2]. When diagnosing SMT-like lesions with a central depression, biopsy specimens should be taken from the depressed region, because most of the lesion is covered by non-tumor mucosa and the cancerous glands are exposed to the surface only in the depressed portion. NBI magnified endoscopy can visualize the obscure mucosal pattern with irregular microvessels in the depressed portion [6]. This pattern is most likely to be in areas of the cancerous glands. Therefore NBI magnified endoscopy can assist in selecting an appropriate biopsy site more precisely than conventional endoscopy.

## Conclusions

There has been no recurrence in this patient in the three years since ER. We succeeded in completely removing the SMT-like duodenal cancer, which appeared to be invading the submucosa or deeper, in a minimally invasive manner by means of ER rather than by performing a pancreaticoduodenectomy. There is a vast difference in quality of life after treatment between ER and pancreaticoduodenectomy. We suggest that SMT-like cancer in the duodenum should be carefully diagnosed, with a view to performing ER, if possible.

## Consent

Written informed consent was obtained from the patient for publication of this case report. A copy of the written consent is available for review by the Editor-in-Chief of this journal.

## Abbreviations

ER, endoscopic resection; EUS, endoscopic ultrasonography; NBI, narrow-band imaging; SMT, submucosal tumor; SR, surgical resection.

## Competing interests

The authors declare that they have no competing interests.

## Authors’ contributions

HI is a doctor in private practice and he introduced the patient to the Department of Surgery at our hospital. KY is a professor in the Department of Surgery who arranged esophagogastroduodenoscopy for preoperative assessment. AD, KG, NY, KS, HT and SS have made substantial contributions to the acquisition and interpretation of clinical data. KG has been involved in drafting the manuscript or revising it critically for important intellectual content. MI provided and revised the pathological data. TK and HT have given final approval for the version to be published. All authors have read and approved the final manuscript.
